# Longitudinal transcriptome analysis reveals distinct gene expression patterns in traditional Chinese medicine syndromes of upper respiratory tract infections

**DOI:** 10.3389/fgene.2024.1483098

**Published:** 2024-11-26

**Authors:** Hao Zou, Changrui Huang, Qinqi Feng, Bang Yu, Jian Liu, Xinyang Shu, Xiaolu Nie, Hongchun Zhang, Xiaohui Zou

**Affiliations:** ^1^ Graduate School of Beijing University of Chinese Medicine, Beijing, China; ^2^ National Center for Respiratory Medicine, National Centre for Integrative Chinese and Western Medicine, State Key Laboratory of Respiratory Health and Multimorbidity, National Clinical Research Center for Respiratory Diseases, Department of Traditional Chinese Medicine for Pulmonary Diseases, China-Japan Friendship Hospital, Institute of Respiratory Medicine, Chinese Academy of Medical Sciences, Beijing, China; ^3^ National Center for Children’s Health, Center for Clinical Epidemiology and Evidence-Based Medicine, Beijing Children’s Hospital, Capital Medical University, Beijing, China; ^4^ National Center for Respiratory Medicine, National Centre for Integrative Chinese and Western Medicine, State Key Laboratory of Respiratory Health and Multimorbidity, National Clinical Research Center for Respiratory Diseases, Department of Pulmonary and Critical Care Medicine, China-Japan Friendship Hospital, Institute of Respiratory Medicine, Chinese Academy of Medical Sciences, Beijing, China

**Keywords:** upper respiratory tract infections, traditional Chinese medicine, wind-cold syndrome, wind-heat syndrome, transcriptome analysis

## Abstract

**Background:**

Wind-cold (WC) and Wind-heat (WH) are common syndromes of upper respiratory tract infections (URTIs) in traditional Chinese medicine (TCM), presenting different clinical features, but the transcriptomic changes associated with these syndromes remained unclear.

**Materials and Methods:**

Patients with WC and WH syndromes were recruited from outpatient unit, pharyngeal swabs were collected for pathogen detection. Peripheral blood samples were obtained on day 1 and day 6, with healthy volunteers as controls. Transcriptome sequencing was performed to identify differentially expressed genes and pathways associated with the two syndromes. Marker genes for each syndrome were identified, and a machine learning classifier was developed.

**Results:**

A total of 124 samples from 34 WC, 30 WH patients, and 16 health controls were included in this study. No significant differences in etiological spectrum were observed between the syndromes. Both syndromes showed distinct gene expression profiles compared to health control. Gene enrichment analysis indicated that TGF-β and Wnt/β -catenin pathways were downregulated in the WH. The oxidative phosphorylation pathways were downregulated in WC cohort compared to the WH cohort. As the URTIs improved from day 1 to day 6, oxidative phosphorylation pathway activity returned to normal levels. The marker genes for WC and WH syndromes were identified and a random forest classifier was built, achieving an accuracy of 0.88.

**Conclusion:**

WC and WH syndromes demonstrated distinct gene expression profiles, supporting more precise TCM diagnosis. WC syndrome is marked by mitochondrial dysfunction, while WH syndrome is characterized by downregulated TGF-β and Wnt/β-catenin pathways.

## Introduction

Upper respiratory tract infections (URTIs) are common diseases and often disrupt daily activities and work (Tang et al., 2017; Yoon et al., 2017). Despite a 40.18% detection rate of respiratory tract pathogens, symptomatic treatment remains the primary option (Zhang et al., 2023a). As a complementary treatment for upper respiratory tract infections, Traditional Chinese Medicine (TCM) has been increasingly recognized for its unique therapeutic benefits (Wu et al., 2021b). A study demonstrated that combining oseltamivir with TCM reduced fever resolution time by 19% compared to oseltamivir alone (Wang et al., 2011). Another study showed that integrating TCM with standard treatment shortened the symptom recovery time in COVID-19 patients from 10 days to 7 days, and significantly reduced the recovery time for fever, fatigue, and cough (Hu et al., 2021).

TCM categorizes URTIs based on patients’ specific symptoms into two syndromes: WC and WH (Jiao et al., 2013). WC syndrome is characterized by fever, a pronounced sensation of chilliness, throat itching without significant pain, and watery nasal discharge. WH syndrome is distinguished by fever without notable chills, sore throat or even a burning sensation, and purulent nasal discharge. The two syndromes are corresponding different host response according to TCM theories. Accurately differential diagnosis of WC and WH syndrome in URTIs is crucial to administer appropriate herbal treatments and ensure effective treatment (You et al., 2021). However, the underlying transcriptomic changes differentiating these two syndromes remain unclear.

Previous studies have revealed several transcriptomic features that characterize different TCM syndromes in other diseases. Lu et al. (2021) identified different gene expression levels among the three TCM syndromes of psoriasis by transcriptome analysis. You et al. (2021) found that the spleen qi deficiency syndrome of chronic atrophic gastritis is characterized by an inflammatory response and the collagen catabolism pathway, and that hsa-miR-122-5p can serve as a marker gene for this syndrome. However, we still know little about the gene expression features of the TCM syndromes in the UTRIs.

In this study, cross-section transcriptomic analysis was used to identify differentially expressed genes between WC and WH syndrome, and their longitudinal variation from disease onset to recovery stage for each syndrome. We identified significant differences in gene expression and enriched pathways between WC and WH syndromes during the initial stage and throughout the course of URTIs. Marker genes for each syndrome were also screened and used to construct effective diagnostic models, which might contribute to precise diagnosis of WC and WH syndromes in URTIs.

## Materials and methods

### Participants recruitment and data collection

This study was reviewed and approved by the ethics committee of China-Japan Friendship Hospital (2023-KY-149). Written informed consent was obtained from all participants. The diagnosis of URTIs followed the guideline for primary care of acute upper respiratory tract infections (2018) (Chinese Medical Association, 2019).

Individuals aged 18 years or older were eligible if they presented within 48 h of the onset of fever (axillary temperature over 37.3°C) and meet the clinical manifestations of WC syndrome or WH syndrome. According to Zhai et al. (2015) and Butler et al. (2020) the median time to symptom relief in patients with upper respiratory tract infections is approximately 6–7 days. Guided by these references, we decided to collect samples on days 1 and 6 to capture transcriptomic changes in the syndrome from onset to recovery. All patients received standard treatment according to guidelines during the study (Chinese Medical Association, 2019). The patient’s condition was assessed using a symptom scoring sheet ranging from 0 to 3 (with higher scores indicating greater severity) for each of the seven symptoms: fever, headache or muscle aches, sore throat, fatigue, runny nose, nasal congestion, and sensation of chilliness; the total symptom score ranged from 0 to 21 (Supplementary Table S1). Healthy volunteers aged 18 and over were recruited from the community, who had not experienced significant symptoms of URTIs or had a history of contact with patients infected with respiratory infectious diseases in the past 4 weeks.

WC syndrome was defined by the presence of at least two of the following symptoms: a conspicuous sensation of chilliness (an extreme sensitivity to cold, where the patient feels cold even in warm environments, despite wearing thick clothing or using heavy blankets), watery nasal discharge, headache or muscle aches, no spontaneous sweating (excessive sweating occurs during the daytime without external triggers, especially during physical activity), and a thin white coating on the tongue (a reference figure provided in Supplementary Figure S1A). In addition, WC syndrome should not manifest as purulent nasal discharge, sore throat, cough with yellow phlegm, or dry and hard stools.

WH syndrome was defined by the presence of at least two of the following symptoms: a sore throat, purulent nasal discharge, spontaneous sweating, headache or muscle ache, and a red tongue with thin yellow coating (a reference figure provided in Supplementary Figure S1B). WH syndrome should not feature a conspicuous sensation of chilliness, nor does it include watery nasal discharge.

### Pharyngeal swab collection, nucleic acid extraction, multiplex RT-PCR, and etiological diagnosis

Upon enrollment, a pharyngeal swab sample was collected from each patient using a sterile swab (Copan, 480°C, Italy). The swab was used to collect samples from the bilateral tongue roots, pharyngeal tonsils, and posterior pharyngeal wall by wiping six times. The sample was then stored at −80°C within 1 h. Nucleic acid extraction was performed using the QIAcube HT kit (Qiagen, Germany).

The Respiratory Six-Item Kit (Shengxiang Biological Technology Co., Changsha, China) was used to detect influenza A virus (Flu A), influenza B virus (Flu B), human rhinovirus (HRV), adenoviruses (Adv), *mycoplasma* pneumonia (MP), and respiratory syncytial virus (RSV). Multiplex RT-PCR was conducted with the following cycling conditions: 50°C for 30 min, followed by 1 min of pre-denaturation at 95°C, and then 45 cycles of 15 s at 95°C and 30 s at 60°C. A sample was considered positive if the Ct value was ≤40. For coronavirus disease 2019 detection, the COVID-19 Detection Kit (Shengxiang Biological Technology Co., Changsha, China) was used. Multiplex RT-PCR was conducted with the following cycling conditions: 50°C for 3 min, followed by 5 s of pre-denaturation at 95°C, and then 41 cycles of 5 s at 95°C and 16 s at 60°C. A sample was considered positive if the Ct value was ≤40.

### Library preparation for transcriptome sequencing

Blood samples were collected on day 1 and day 6, then stored at −80°C in PAXgene Blood RNA tubes (Qiagen, Germany) for later analysis. Total RNA was isolated using the standard procedures of the PAXgene Blood RNA Kit (Qiagen, Germany). RNA integrity was verified using the RNA Nano 6000 Assay Kit on an Agilent Bioanalyzer 2,100 system (Agilent Technologies, CA, United States).

mRNA was isolated from the total RNA using poly-T oligo-attached magnetic beads. Fragmentation was induced using divalent cations at elevated temperatures in First Strand Synthesis Reaction Buffer (5X). The first strand of cDNA was synthesized with a random hexamer primer and M-MuLV Reverse Transcriptase, followed by RNA degradation with RNaseH. The second strand of cDNA was synthesized using DNA Polymerase I and dNTPs. Overhangs were smoothed to blunt ends through the activities of exonuclease and polymerase. After adenylation of the 3′ends of DNA fragments, adaptors with a hairpin loop structure were ligated to prepare for hybridization. To select cDNA fragments of the desired length, specifically 370–420 bp, the library fragments were purified using the AMPure XP system (Beckman Coulter, Beverly, United States) and subjected to PCR amplification to generate the final library.

The constructed library was initially quantified using a Qubit 2.0 Fluorometer (Life Technologies, United States) and diluted to a concentration of 1.5 ng/μL. The insert size was assessed using the Agilent 2,100 Bioanalyzer to ensure it met the required specifications. Following this, qRT-PCR was employed to precisely quantify the effective concentration of the library, thereby confirming its quality. Finally, the various libraries were pooled together and sequenced on an Illumina NovaSeq 6,000 system (Illumina, United States) utilizing a 150bp paired-end sequencing approach.

### Differential expression analysis and enrichment analysis

The raw data, in fastq format, were evaluated using FastQC (version 0.11.5) to assess data quality. Subsequently, the raw data were filtered using fastp (version 0.21.0) to remove low-quality reads, adapter sequences, low-complexity sequences, and rRNA sequences, resulting in clean data. The clean data were then mapped to the GRCh38 reference human genome using Hisat2 (version 2.2.1). FeatureCounts (version 1.6.3) was used to count the number of reads mapped to each gene. Subsequently, the counts per million reads (CPM) values for each gene were calculated.

Differential expression analysis was performed using the edgeR package (version 1.10.1) (Robinson et al., 2010). *p*-value <0.05 and fold change >2 were set as threshold to identify genes with significant differential expression (Li et al., 2021; Lu et al., 2021; Zhang et al., 2023b). For these differentially expressed genes, enrichment analysis was performed using the Kyoto Encyclopedia of Genes and Genomes (KEGG) database and Gene Ontology (GO) biological processes database (Kanehisa et al., 2017; The Gene Ontology, 2019). Additionally, we performed Gene Set Enrichment Analysis (GSEA) analysis to analyze the pathways from the hallmark gene sets in MSigDB (Subramanian et al., 2005). Statistical analyses were performed using two-sided Mann-Whitney U tests. All statistical analyses were conducted using R software (version 4.3.2).

### Weighted gene co-expression network analysis (WGCNA)

To minimize noise from genes with low variability across samples and enhance the accuracy of downstream analyses, we employed WGCNA on the top 5,000 genes ranked by median absolute deviation (Jianping et al., 2022; Bai et al., 2023). This approach aims to identify gene modules associated with Wind-Cold or Wind-Heat syndromes.

The cluster analysis of the samples was initially conducted using the hclust function, with an appropriately chosen threshold value to detect and eliminate outliers, in line with the methods of WGCNA (version 1.72.1) (Langfelder and Horvath, 2008). The selection of the soft thresholding power β-value was facilitated by the pickSoftThreshold function, aiming to attain a scale-free topology criterion of 0.85. Upon identifying the optimal power value, the WGCNA algorithm was applied to construct co-expression networks, also known as modules, ensuring a minimum module size of 30. These modules were delineated as branches on a hierarchical clustering dendrogram and each was distinctly marked with a unique color label. Subsequently, the genes in the module of interest were analysed for KEGG enrichment using the ClusterProfiler R software package (version 4.10.0) (Wu et al., 2021a). To visualize the networks of the target modules and pinpoint the top ten hub genes, Cytoscape software (version 3.10.1) alongside the cytoHubba plugin (version 0.1) was employed.

### Immune infiltration analysis

The CIBERSORT package (version 0.1.0) was used to predict the proportions of 22 immune cell types in peripheral blood (Chen et al., 2018), including naïve B cells, memory B cells, plasma cells, CD8 T cells, naïve CD4 T cells, resting and activated memory CD4 T cells, Tregs, γδT cells, resting and activated natural killer cells, monocytes, M0, M1, and M2 macrophages, resting and activated dendritic cells, resting and activated mast cells, eosinophils, and neutrophils. The aim was to explore whether there was a significant difference in the composition of immune cells in peripheral blood between the WC syndrome and WH syndrome on days 1 and 6.

### Constructing a diagnostic model

A random forest model was constructed using the gene expression data of the selected genes, with the scikit-learn library (version 1.3.0) in Python 3.11. The optimal parameter combination for the random forest model was determined using the GridSearchCV function, which performed an exhaustive search over specified parameter values. The average score of cross-validation was calculated using the cross_val_score function, whereas the cross-validation prediction results were obtained using cross_val_predict. The roc_curve and auc functions from the scikit-learn library were employed to plot the receiver operating characteristic (ROC) curve and calculate the area under the curve (AUC).

## Results

### Participants’ characteristics

A total of 80 participants were included in the study: 34 with WC syndrome, 30 with WH syndrome, and 16 healthy volunteers. Among patients with WC syndrome, there were 18 males (52.94%) and 16 females (47.06%), with a median age of 42.5 years (IQR: 29.75–54) and a BMI of 22.2 (IQR: 20.8–23.12). For those with WH syndrome, there were 10 males (33.33%) and 20 females (66.66%), with a median age of 35 years (IQR: 32–38.75) and a BMI of 23.5 (IQR: 21.78–24.88). On day 1, the mean symptom scores were 6 (IQR: 6–7) for the WC syndrome group and 7 (IQR: 5–8) for the WH syndrome group, with no significant differences observed. Blood count quantification showed no significant differences between the two groups in terms of erythrocytes, leukocytes, hemoglobin, and platelet counts. The percentages of neutrophils and lymphocytes also did not differ significantly.

Sixteen healthy volunteers were recruited from the community for this study, consisting of 11 males (68.75%) and 5 females (31.25%), with an average age of 29 years (IQR: 28–40.25) and a mean BMI of 22.9 (IQR: 21.23–24.55). There were no significant differences in sex distribution, age, or BMI between the healthy volunteers and the WC and WH syndrome groups. Detailed information can be found in Table 1.

**TABLE 1:** Baseline characteristics and laboratory parameters of participants.

Characteristics	WC (n = 34)	WH (n = 30)	Health (n = 16)	*p*-value
Age (year)	42.5 (29.75,54)	35 (32,38.75)	29 (28,40.25)	0.056
Sex				
Male, n (%)	18 (52.94%)	10 (33.33%)	11 (68.75%)	0.059
Female, n (%)	16 (47.06%)	20 (66.66%)	5 (31.25%)	
Height (cm)	171 (161.5,176)	164.5 (160,170)	169.5 (166.75,175.25)	0.070
Weight (kg)	65.5 (58.5,70)	65 (53.5,70)	66 (56.65,76.25)	0.810
BMI (kg/m^2^)	22.2 (20.8,23.18)	23.5 (21.78,24.88)	22.9 (21.23,24.55)	0.144
Temperature (°C)	38.1 (38,38.3)	37.65 (37.6,37.7)	36.4 (36.18,36.43)	2.9e-12
Total symptom score on day 1	6 (6,7)	7 (5,8)		0.429
Total symptom score on day 6	0 (0,0)	0 (0,0)		0.775

Symptoms were fever, headache or muscle aches, sore throat, fatigue, runny nose, nasal congestion and sensation of chilliness. Each symptom was scored as 0 (none), 1 (mild), 2 (modest), or 3 (severe). The summed scores of all symptoms on day 1 or day 6 are shown. Abbreviations: BMI, body mass index.

Between the three groups, the gender share was tested using the chi-square test. For the other characteristics, at least one group did not fit the normal distribution, a Mann-Whitney *U* test is employed, and the results are presented as median and interquartile range (IQR).

Etiological diagnosis showed that no patients in either group tested positive for Flu B, HRV, Adv, MP, or RSV. Only one patient in the WH syndrome group tested positive for Flu A. Ten patients in the WC syndrome group and one patient in the WH syndrome group tested positive for COVID-19, with a *p*-value less than 0.05 (Table 2).

**TABLE 2:** Etiological diagnosis and complete blood count of participants.

Etiological diagnosis	WC (n = 34)	WH (n = 30)	*p*-value
Flu A	0	1	0.950
Flu B	0	0	1
HRV	0	0	1
Adv	0	0	1
MP	0	0	1
RSV	0	0	1
COVID-19	10	1	0.015

Complete blood count	WC(n = 33)	WH(n = 28)	*p*-value
Erythrocytess (×10^12^/L)	4.52 ± 0.59	4.78 ± 0.52	0.066
Leukocytess (×10^9^/L)	6.77 (5.85,9.09)	7.02 (5.61,9.62)	0.794
Hemoglobins (g/L)	133 (119,153)	146.5 (126,155)	0.478
Plateletss (×10^9^/L)	253.33 ± 45.02	251.14 ± 46.79	0.920
Neutrophils (%)	60.53 ± 4.18	58.26 ± 6.57	0.122
Lymphocytes (%)	31.01 ± 4.64	32.81 ± 5.66	0.185

For etiological diagnosis, the Fisher exact testing was used for intergroup comparisons. For Complete Blood Count, if both groups of compared data follow a normal distribution, an independent samples *t*-test is used, and the results are displayed as mean ± standard deviation. If one or both groups do not adhere to a normal distribution, a Mann-Whitney *U* test is employed, and the results are presented as median and interquartile range (IQR).

Abbreviations: Flu A Influenza A virus, Flu B Influenza B virus, HRV, human rhinovirus, Adv adenovirus; MP, *mycoplasma*; RSV, respiratory syncytial virus; COVID-19, Coronavirus Disease 2019.

Due to the absence of complete blood count results in one patient with WC, syndrome and in two patients with WH, syndrome, the sample size for complete blood count in WC, syndrome is 33 and in WH, syndrome is 28.

**FIGURE 1:**
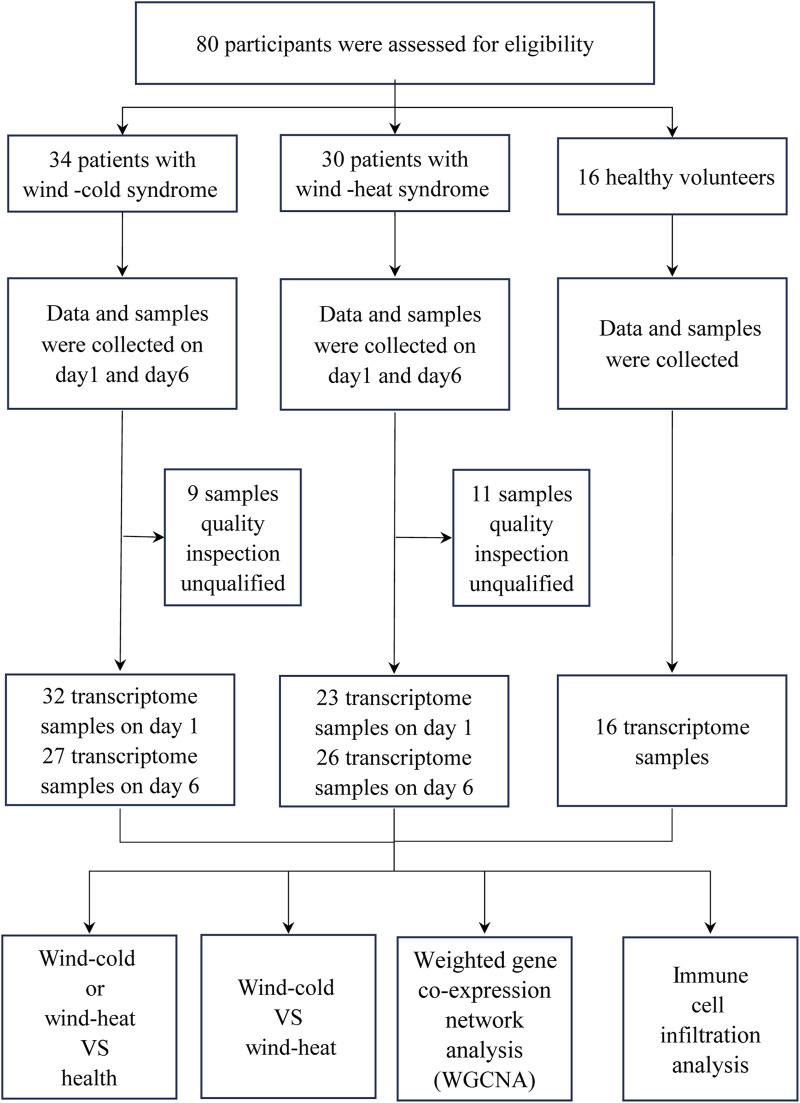
Flow chat of the study.

Following RNA extraction and library construction, 20 samples were excluded due to quality inspection unqualified. Consequently, 124 transcriptome samples were included in the analysis, comprising 32 and 27 samples of WC syndrome patients on day 1 and day 6, respectively; 23 and 26 samples of WH syndrome patients on day 1 and day 6, respectively; and 16 samples of healthy volunteers (Figure1). In subsequent analyses, we will collectively compare samples from day 1 and day 6 of WC syndrome, day 1 and day 6 of WH syndrome, and healthy control group.

### WC and WH syndromes exhibited similar elevated pathways compared to health controls on day 1

**FIGURE 2:**
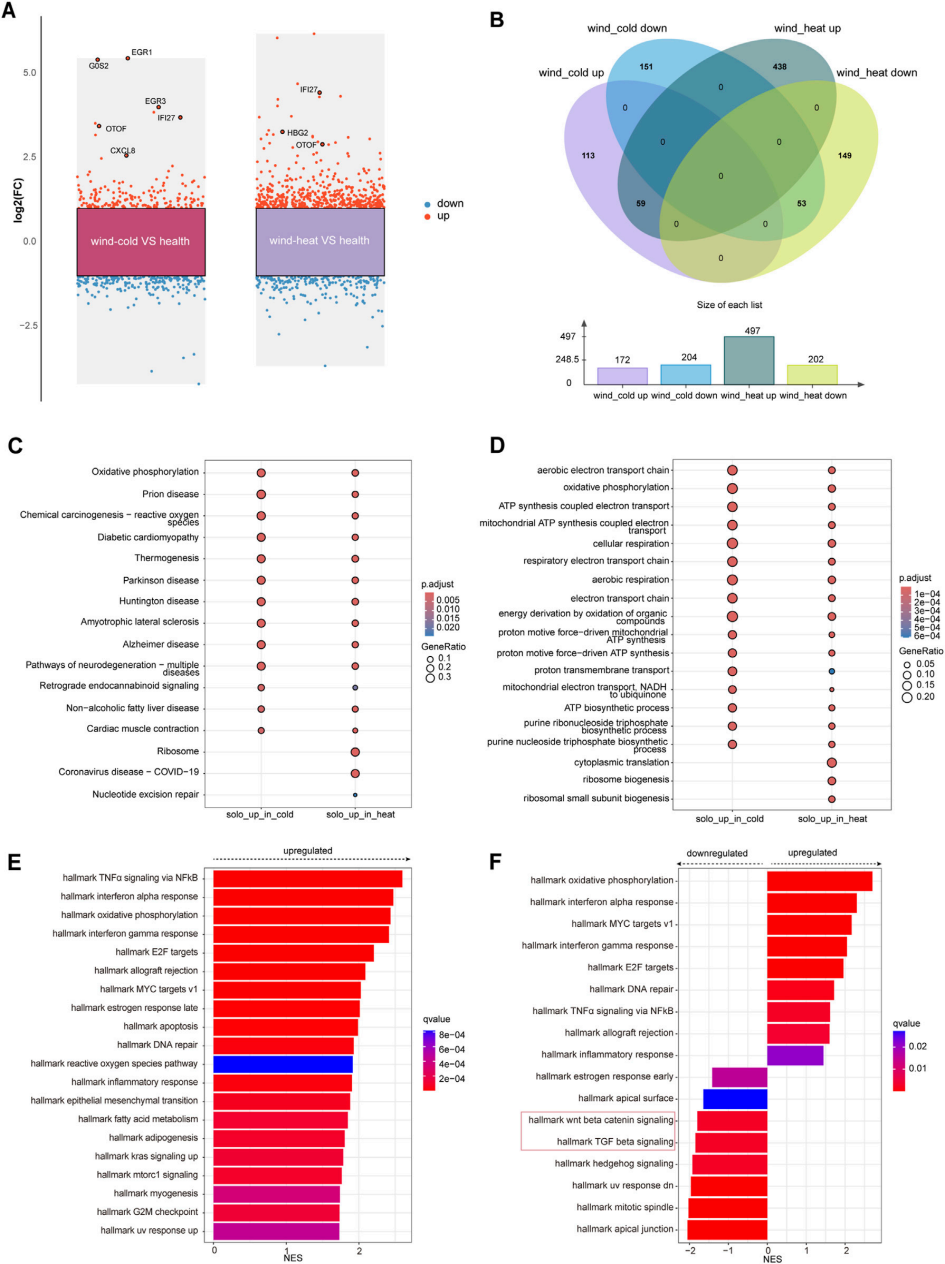
Differential expressed genes analysis among WC, WH, and the healthy groups on day 1. **(A)** Differentially expressed genes (red: upregulated, blue: downregulated) between each syndrome vs. healthy, on day 1. **(B)** Venn diagram of differentially expressed genes on day 1 between syndromes and healthy. **(C)** KEGG pathways for upregulated genes in each syndrome on day 1. **(D)** Top 10 GO pathways for upregulated genes in each syndrome on day 1. **(E)** Top 20 pathways analysed by GSEA of differentially expressed genes for WC vs. healthy on day 1. **(F)** GSEA of differentially expressed genes for WH vs. healthy on day 1.

On day 1, when compared to the healthy control, 175 genes were upregulated and 206 genes were downregulated in the WC syndrome, while 501 genes were upregulated and 203 genes downregulated in the WH syndrome (Figure 2A; Table 3). To evaluate whether the differentially expressed genes in the WC group were similar to those in the WH group compared to the healthy group, a Venn diagram was created, illustrating both upregulated and downregulated genes from each comparison. The diagram revealed a majority of distinct genes between the two groups, with only a minor overlap (Figure 2B).

**TABLE 3:** Top 20 differentially expressed genes between wind-cold or wind-heat syndromes and healthy group.

Comparisons	Up	Down	Top20 upregulated genes	Top20 downregulated genes
Wind-Cold day1 VS health	175	206	EGR1, G0S2, EGR3, FOSB, IFI27, OTOF, MTND1P23, EGR2, PNMA6A, CXCL8, NKX3-1, MIR12136, OSM, TWIST2, CCL23, ND6, RPS14P1, AREG, RPL13AP20, HBEGF.	RPS23P9, ARHGEF33, HBG1, PPEF2, LOC101927543, TRAJ7, PARD3B, CD177, ZKSCAN7-AS1, AP3B2, P2RY4, MIR4462, MTND4LP23, LINC01980, KRT8P43, RPL9P7, ZSCAN23, LINC02751, KLHL25P1, TRAJ4
Wind-Heat day1 VS health	501	203	MTND1P23, IFI27, RPL17P39, RPL7AP62, RPL26P32, RPL14P3, RPL18AP6, RPL7AP66, RPL17P6, RPL37P6, TMSB4XP4, HBG2, RPL5P4, RPL26, RPS10P13, RPS10P3, RPL13AP25, OTOF, RPL39, RPL21P16	LRRC26, EEF1DP7, GPR84-AS1, FGD1, CNTNAP3, CNTNAP3C, DOCK4, LINC00304, TUBB1, RPS2P51, SULT1A2, IGHV4-61, TALAM1, RN7SL381P, CD300LD, IGHV3-13, SNORA38, CYLD-AS2, MMP19, KCNG2
Wind-Cold day1 VS day6	44	38	RAP1GAP, FAM3B, ERFE, OTOF, IFI27, DEFA1, ESRG, VSTM2B, GYPA, IGKV1D-13, SLC6A19, IGF2, ITGA2B, BCAM, BOLA2, BOLA2B, LOC124907841, DNAH2, ND6, DDX11L16	HBG2, RPL26P32, RPL36AP37, RPL17P39, LOC100507336, OLAH, RPL34, RPL26P19, RPL17P18, UQCRB, EFHB, RPS24, RPL17P6, RPL26P4, COX6C, PET117, BLVRBP1, RPL34P18, PFDN4, RPL17P7
Wind-Cold VS Wind-Heat on day1	92	348	EGR1, CES3, MTCO1P12, G0S2, FOSB, C4BPA, NKX3-1, RPL21P19, HBA1, RPL21P134, FIBCD1, SCN11A, MYOM2, SLX1B, LOC124907837, BCAM.	RPL26P32, ARHGEF33, RPL17P39, PPEF2, TMSB4XP4, RPL7AP62, RPL14P3, RPS10P13, RPS10P3, RPL26P4, MTND1P23, RPL5P4, RPL23P6, RPL26, RPL17P6, RPL7AP66

The threshold for all differential analysis schemes is set at FC (fold change) = 2 and *p*-value = 0.05.

KEGG and GO enrichment analyses of the genes uniquely upregulated in either WC or WH syndromes demonstrated that many of these gene sets are enriched in similar pathways. However, certain pathways, such as ribosome biogenesis and cytoplasmic translation, were exclusively enriched among genes upregulated in the WH syndrome (Figures 2C, D). In contrast, KEGG and GO enrichment analyses of genes downregulated in both groups did not identify any significantly enriched pathways.

For genes that were differentially expressed in both syndromes, KEGG enrichment analysis highlighted pathways commonly associated with upper respiratory infections, including the IL-17 signaling pathway, viral proteins interactions with cytokine and cytokine receptors, and the COVID-19 signaling pathway (Supplementary Figure S2A). GO enrichment analysis further indicated activation of pathways like response to viruses, while pathways such as import into nucleus were suppressed in both syndromes (Supplementary Figure S2B).

To prevent missing changes in certain gene sets due to subtle alterations in individual genes from threshold-based filtering, GSEA was conducted on the differential gene sets between WC and healthy groups, as well as WH and healthy groups, on day 1. The GSEA revealed that pathways such as TNFα signaling via NF-κB, oxidative phosphorylation, and interferon alpha response were activated in the WC syndrome, with no pathways found to be suppressed (Figure 2E; Supplementary Figure S4). In contrast, the WH syndrome showed activation of oxidative phosphorylation and interferon alpha response pathways, but also showed downregulation of the Wnt/β-catenin signaling and TGF-β signaling pathways when compared to the healthy group (Figure 2F).

### Significant difference in gene expression profiles between WC syndrome and WH syndrome on day 1

**FIGURE 3:**
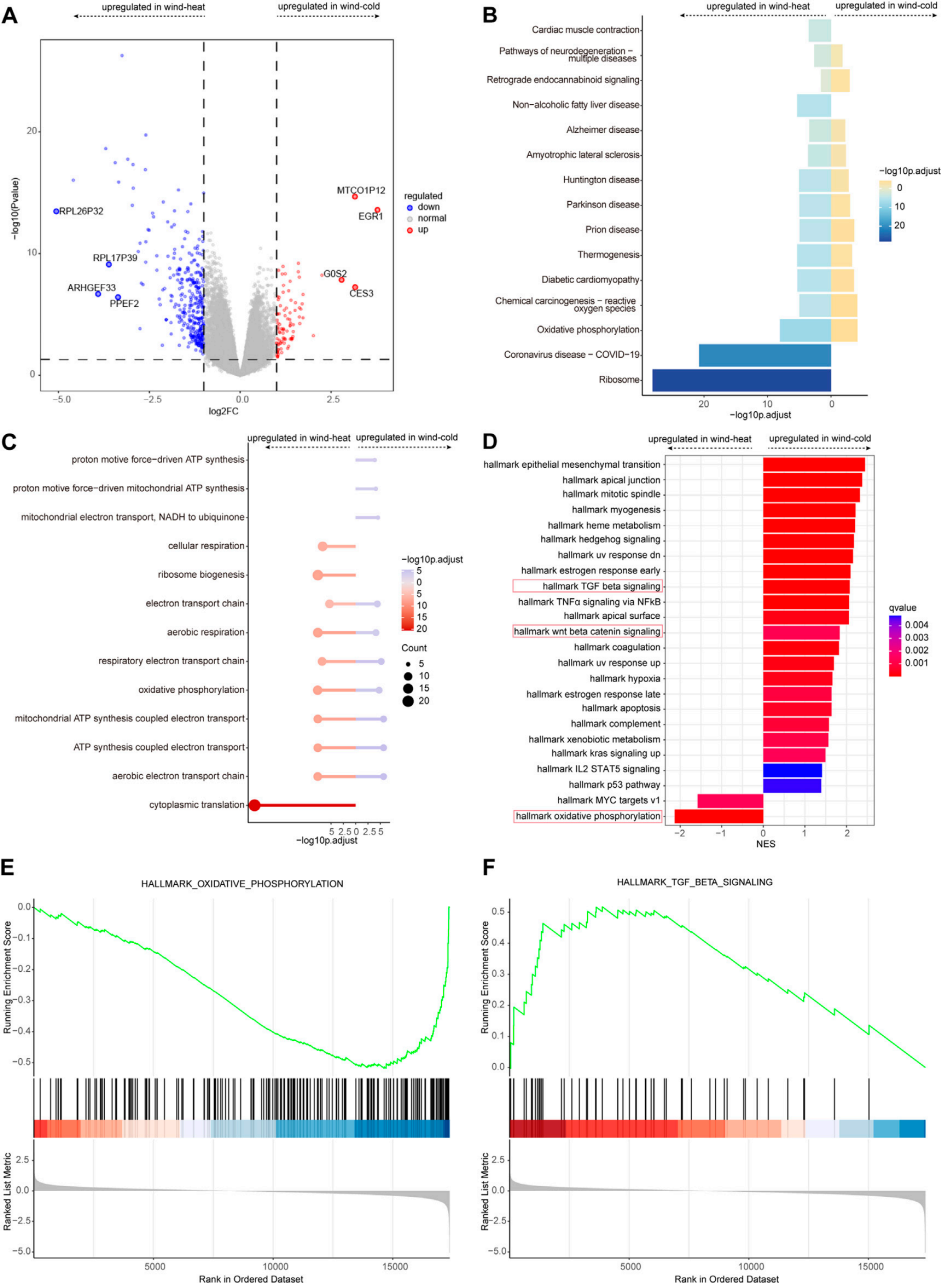
Differential expressed genes analysis between WC and WH syndromes on day 1. **(A)** Volcano plot of differentially expressed genes for WC vs. WH. **(B)** KEGG pathways for differentially expressed genes for WC vs. WH. **(C)** GO pathways for differentially expressed genes for WC vs. WH. **(D)** GSEA for differentially expressed genes for WC vs. WH. **(E)** Curves of GSEA enrichment scores for oxidative phosphorylation in WC vs. WH on day 1. **(F)** Curves of GSEA enrichment scores for TGF-β signaling in WC vs. WH on day 1.

The samples collected on day 1 were used to profile the transcriptomic changes related to the two syndromes, as the syndrome features were prominent at the early stage of disease. Compared to WH syndrome, there were 92 genes upregulated and 348 genes downregulated in WC syndrome on day 1 (Figure 3A). KEGG enrichment analyses of the up- and downregulated genes showed that these genes were enriched in many of the same pathways, such as thermogenesis and the oxidative phosphorylation pathway. Pathways including cardiac muscle contraction, non-alcoholic fatty liver disease, coronavirus disease—COVID-19, ribosome pathway were solely enriched in the WH groups (Figure 3B).

GO enrichment analysis revealed similar enrichment patterns, that most of the up- and downregulated genes were enriched in the same pathways; in addition, pathways such as proton motive force-driven ATP synthesis were enriched only in WC group, and the cytoplasmic translation pathway was enriched only in WH group (Figure 3C).

GSEA enrichment analysis revealed that Wnt/β-Catenin signaling pathway, TNFα signaling via NFκB pathways (Figure 3D), and TGF β signaling pathway (Figure 3E) appeared only in WC group, whereas oxidative phosphorylation (Figure 3F) and MYC targets v1 pathways were enriched in WH group.

### Longitudinal gene expression variation from day 1 to day 6 was different between WH and WC syndromes

**FIGURE 4:**
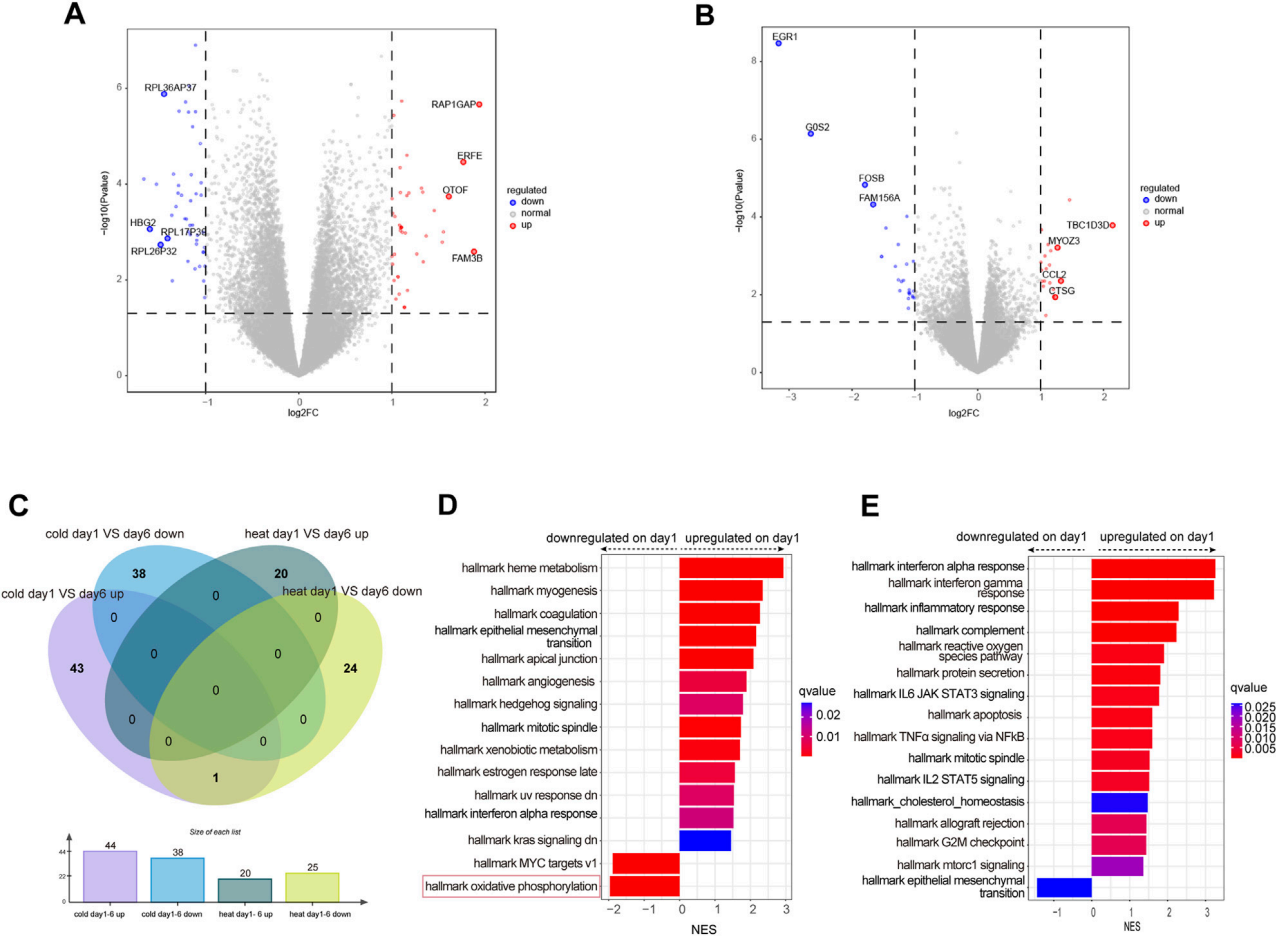
Differential expressed genes analysis between day 1 and day6 in WC or WH syndrome. **(A)** Volcano plot of differentially expressed genes between day 1 and day 6 in WC syndrome. **(B)** Volcano plot of differentially expressed genes between day 1 and day 6 in WH syndrome. **(C)** Venn diagram of differentially expressed genes on day 1 compared to day 6 in WC syndrome versus those in WH syndrome. **(D)** GSEA of differentially expressed genes between day 1 and day 6 in WC syndrome. **(E)** GSEA of differentially expressed genes between day 1 and day 6 in WH syndrome.

To determine the gene expression variation throughout the entire disease stage in WC and WH patients, we identified the differentially expressed genes for each syndrome between day 1 and day 6. In the WC group, there were 44 genes upregulated on day 1, including HBG2 and RPL36AP37, and 38 genes downregulated, such as OTOF and ERFE (Figure 4A; Table 3). In the WH group, there were 25 genes upregulated on day 1, including EGR1 and G0S2, and 20 genes downregulated, including TBC1D3D and CCL2 (Figure 4B; Table 3). Venn diagrams then showed no overlap in differentially expressed genes between day 1 and day 6 of WC and WH syndrome, except for one pseudogene, RPL34P18 (Figure 4C).

Compared to day 6, genes upregulated on day 1 in WC syndrome are primarily associated with the positive regulation of cellular response to insulin stimulus and protein maturation by iron-sulfur cluster transfer pathways, while downregulated genes are related to the cytoplasmic translation pathway, as identified through GO enrichment analysis (Supplementary Figure S2C). GSEA indicates that on day 1 of WC syndrome, pathways such as interferon alpha response and heme metabolism are upregulated, whereas oxidative phosphorylation and MYC targets v1 are downregulated (Figure 4D).

In the WH syndrome, genes upregulated on day 1 are enriched in several immune-related pathways, such as humoral immune response, defense response to virus, and response to molecules of bacterial origin (Supplementary Figure S2D). GSEA also shows an upregulation in multiple immune pathways, such as interferon alpha response, inflammatory response, and reactive oxygen species pathways (Figure 4E).

### Identification of gene modules closely related to WC or WH syndromes

**FIGURE 5:**
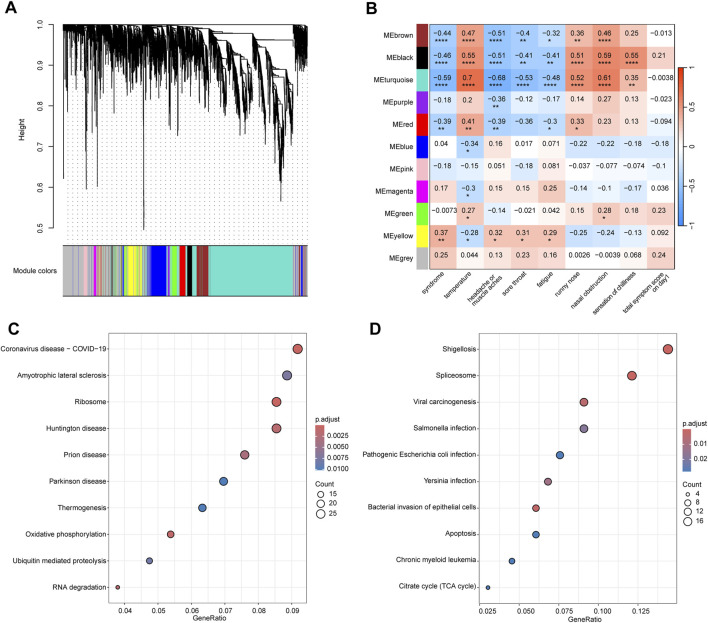
WGCNA analysis of the top 5,000 genes with highest median absolute deviation in WC and WH Syndromes. **(A)** Cluster dendrogram of the top 5,000 genes in WC syndrome and WH syndrome on day 1. **(B)** Module-trait relationships between 11 gene modules and clinical traits. Numbers indicate the size of the correlation, positive values represent positive correlation, the larger the value, the redder the module colour; negative values represent negative correlation, the smaller the value, the bluer the module colour. *p-value < 0.05; **p-value < 0.01; ***p-value < 0.001; ****p-value < 0.0001. **(C)** Top 10 pathway of KEGG enrichment for genes in the brown, black, blue, and turquoise modules. **(D)** Top 10 pathway of KEGG enrichment for genes in the pink module.

We conducted WGCNA analysis on the top 5,000 genes ranked by median absolute deviation on day 1 in 32 patients with WC syndrome and 23 patients with WH syndrome, along with the clinical traits of these patients. Based on the dissimilarity measure of the topological overlap matrix (1-TOM), hierarchical clustering analysis identified 11 modules (Figure 5A).

We found that genes within the brown, black, and turquoise (*p* < 0.001) modules, as well as the red module (*p* < 0.01), were significantly negatively correlated with the syndrome, indicating that the expression of these genes is suppressed in cases of Wind-Cold syndrome (Figure 5B). KEGG analysis revealed that genes in these modules are primarily associated with pathways related to COVID-19, Huntington’s disease, and oxidative phosphorylation (Figure 5C). The top 10 hub genes selected from these modules are RPS3, RPS16, RPL11, RPL5, RPS18, RPS9, RPS27A, RPS6, RPS5, and RPS14, all of which encode ribosomal proteins that are crucial for the process of protein synthesis (Supplementary Figure S3A).

Moreover, genes in the yellow module were significantly positively correlated with the syndrome (*p* < 0.01), indicating that the upregulation of these genes is strongly associated with WH syndrome. KEGG enrichment analysis revealed that genes in these modules are primarily associated with pathways such as viral carcinogenesis, pathogenic *Escherichia coli* infection, bacterial invasion of epithelial cells, and apoptosis (Figure 5D). Through analysis, the top ten hub genes identified from the yellow module are HNRNPK, VCP, BRD4, ACTB, EFTUD2, ENO1, TP53, TRIM28, POLR2A, and PRPF8 (Supplementary Figure S3B). Functionally, HNRNPK, BRD4, EFTUD2, POLR2A, and PRPF8 are involved in mRNA synthesis and processing. ACTB is a crucial component of the cytoskeleton. VCP and TP53 play roles in cell cycle control and DNA repair processes.

### WC and WH syndromes showed different immune cell activation

**FIGURE 6:**
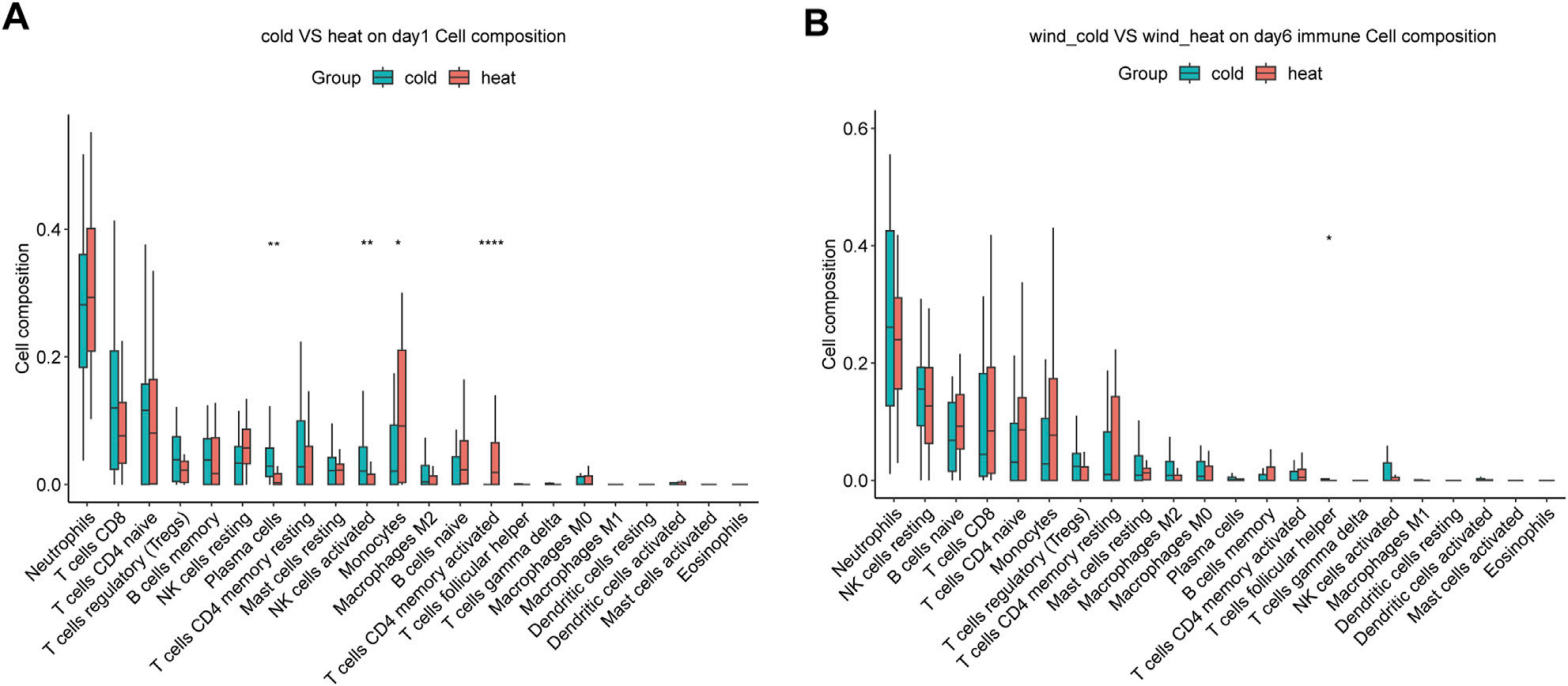
Immune infiltration analysis between WC and WH syndromes. **(A)** Immune infiltration analysis between two group on day 1. **(B)** Immune infiltration analysis between two group on day 6. *p-value < 0.05; **p-value < 0.01; ****p-value < 0.0001.

We compared the immune cell composition in peripheral blood between WC and WH syndromes on day 1 and day 6, using the abundances of 22 cell types estimated by CIBERSORT algorithm. On day 1, plasma cells and activated NK cells were more abundant in the WC syndrome, while monocytes and activated CD4 memory T cells were more prevalent in the WH syndrome. Other immune cell types did not differ significantly between the two groups (Figure 6A). By day 6, the proportions of immune cells were generally similar, except for follicular helper T cells, which remained more abundant in the WC syndrome (Figure 6B).

### Identification of syndrome related gene marker and establishment of machine learning classifier for syndrome differentiation

To identify potential marker genes for WC syndrome, which must exhibit significant differences from the healthy group, WH day 1, and WC day 6. Therefore, a Venn diagrams was constructed to identify shared genes among three sets of differentially expressed genes: genes differentially expressed between WC syndrome and the healthy group on day 1, genes differentially expressed between WC syndrome and WH syndrome on day 1, and genes differentially expressed between day 1 and day 6 for WC syndrome. A similar approach was applied to WH syndrome.

**FIGURE 7:**
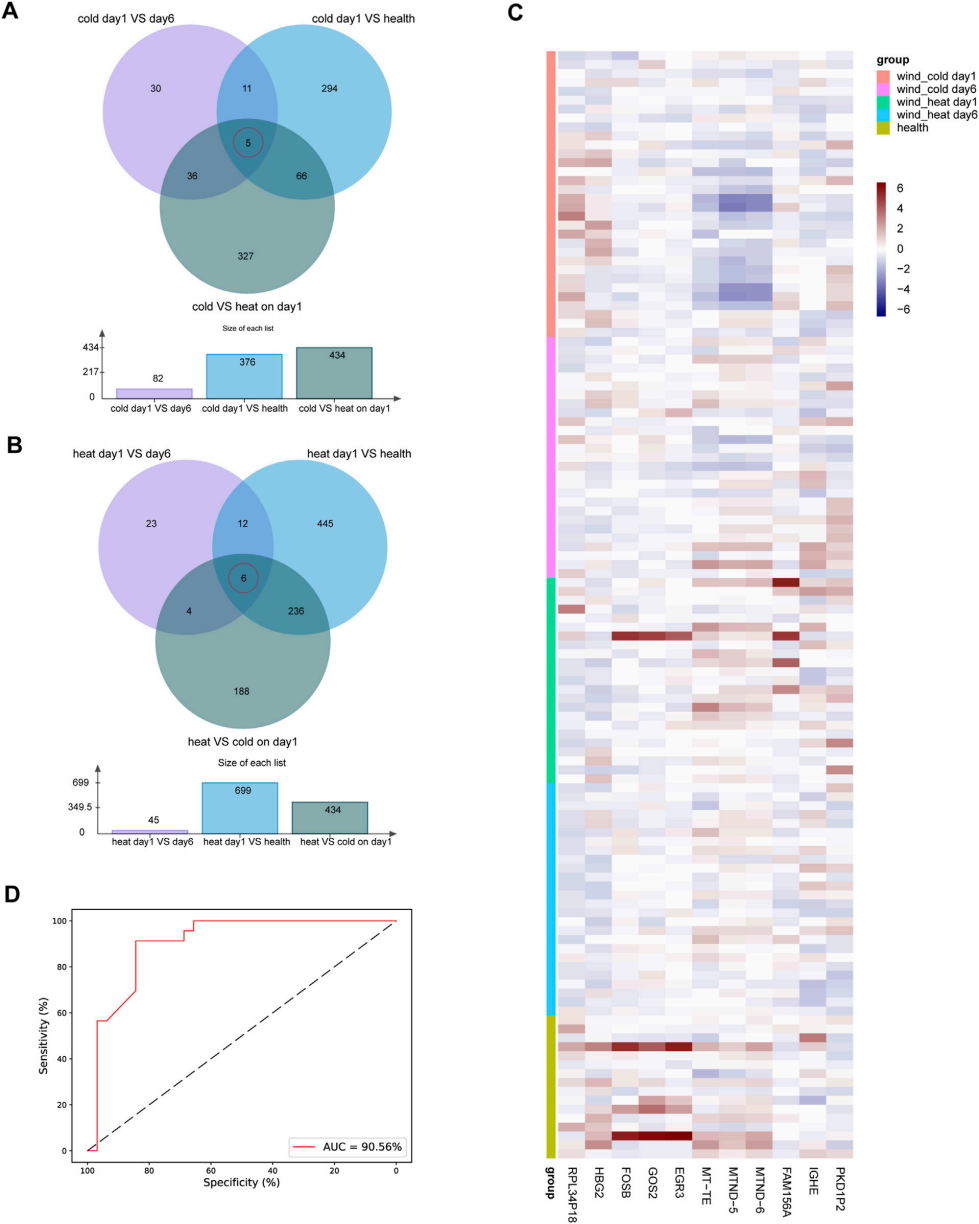
Biomarker genes in WC syndrome or WH syndrome. **(A)** The Venn diagram of differentially expressed genes between day 1 and day 6 in WC syndrome, between day 1 of WC syndrome and the healthy group, and between WC syndrome and WH syndrome on day 1 shows 5 biomarker genes in WC syndrome: IGHE, HBG2, MT-ND5, MT-ND6, and MT-TE. **(B)** The Venn diagram of differentially expressed genes between day 1 and day 6 in WH syndrome, between day 1 of WH syndrome and the healthy group, and between WC syndrome and WH syndrome on day 1 shows 6 biomarker genes in WH syndrome: EGR3, GOS2, FAM156A, RPL34P18, PKD1P2, and FOSB. **(C)** Heatmap of the expression levels of 11 potential biomarker genes in different groups. **(D)** ROC curves for the random forest diagnostic model.

In the WC syndrome, five potential biomarker genes were discovered: IGHE, HBG2, MT-ND5, MT-ND6, and MT-TE (Figure 7A; Supplementary Figure S6). In the WH syndrome, we identified six potential biomarker genes: EGR3, GOS2, FAM156A, RPL34P18, PKD1P2, and FOSB (Figure 7B; Supplementary Figure S6). Furthermore, based on the gene expression data, we generated a heatmap of these 11 genes to elucidate the difference in expression levels among different groups (Figure 7C).

These findings led us to investigate the potential of these genes for accurately diagnosing WC or WH syndromes. To test this hypothesis, a random forest model was developed. The training set comprised CPM values of 11 marker genes from 32 WC syndrome patients and 23 WH syndrome patients. Parameter tuning was conducted using GridSearchCV on this dataset, employing 10-fold cross-validation to evaluate combinations of n_estimators, max_depth, max_features, and min_samples_split. The data was structured with “syndrome” as the target variable, while the remaining columns were used as features.

Through parameter optimization, the optimal combination was determined: n_estimators at 22, max_depth at 5, max_features at 3, and min_samples_split at 2. The model achieved an average accuracy of 0.88 during cross-validation. Using this optimized model, further cross-validation on the entire dataset was performed to compute and plot the ROC curve with the corresponding AUC, which reached 90.56%. This result demonstrates the model’s strong performance in diagnosing WC and WH syndromes. Sensitivity and specificity on the ROC curve were expressed as percentages for clearer performance visualization (Figure 7D).

## Discussion

Traditional Chinese medicine is an effective complementary therapy for upper respiratory tract infections, developing a unique set of diagnostic and therapeutic protocols (Wu et al., 2023). Accurately distinguishing URTIs into different syndromes and selecting appropriate treatment plans are crucial steps in the practice of TCM for URTIs. For example, Gui Zhi Jia Ge Gen Tang is specifically indicated for the WC syndrome, while Chaiqin Qingning capsules are more suitable for the WH syndrome (Wan et al., 2022; Xu et al., 2022). Exploring the pathological differences between the two syndromes would help identify potential targets for TCM and objective diagnostic biomarkers.

WC syndrome and WH syndrome showed no difference on etiology spectrum in this study. Likewise, a study involving 88 cases of H1N1 influenza, 1,083 cases of H3N2 influenza, and 278 cases of influenza B found no differences in the syndromes caused by these three pathogens (Lu et al., 2020). Moreover, a meta-analysis of 4,367 influenza patients showed no difference in the proportion of WC and WH syndromes between influenza A and influenza B (Lei et al., 2023). Furthermore, another meta-analysis of 2,139 COVID-19 patients’ TCM syndrome distribution indicated that, despite being infected with the same pathogen, patients could be classified into different syndromes, with cold and heat being the most prevalent types (Li et al., 2020). These findings imply that the symptom differences between WC and WH syndromes are unlikely to be directly related to the specific pathogens causing the infections. The sensitivity analysis also showed that the presence of COVID-19 does not have a significant impact on gene expression patterns in both WC and WH syndromes.

Transcriptomic analysis can provide a detailed landscape of gene expression in patients at specific time points. In this study, we conducted the first longitudinal transcriptomic analysis to explore the similarities and differences in biological response mechanisms of WC syndrome and WH syndrome in URTIs, focusing on host gene expression changes. Compared with the results of other transcriptomic studies of upper respiratory tract infections, the trends of changes in multiple pathways in the present study are consistent (Supplementary Table S2) (Herberg et al., 2013; Rosa et al., 2021; Nicoletti et al., 2022), e.g., upregulation of interferon and NF-κB pathways, and downregulation of the TGF-β pathway, which suggests that the present results are reliable.

We found that several pathways, such as the interferon alpha response, TNFα/NF-κB signaling, and IL-17 signaling pathways, are enriched in both WC and WH syndromes. Although in Chinese medicine theory, cold syndrome and heat syndrome are opposite relationships, there are many similarities in the pathways enriched for differential gene enrichment in WC and WH syndromes. These shared enriched pathways suggest some common pathological changes between the two syndromes of upper respiratory tract infections, which may be common therapeutic targets for TCM. In line with this, it has been found that Chinese herbal medicines applicable only to WC syndrome or WH syndrome can exert therapeutic effects by inhibiting IL-17 signaling pathway or TNFα/NF-κB signaling pathway (Ma et al., 2020; Tang et al., 2022; Wang et al., 2022; Li et al., 2023). However, we also found gene expression changes specific to both syndromes.

During upper respiratory tract infections, cellular metabolic demands rise to support various immune responses (Keshavarz et al., 2020). Correspondingly, the oxidative phosphorylation pathway is activated in both WC and WH syndrome. However, compared to WH syndrome, oxidative phosphorylation is found to be downregulated in WC syndrome, indicating the possibility of mitochondrial dysfunction in WC syndrome. Previous studies have shown that this pathway may be inhibited during infections with various viruses (Gibbs et al., 2003; Guarnieri et al., 2023). Mahuang Tang, a representative formula for treating WC syndrome, has been shown to increase the oxygen consumption and activity of ATPase in mice (Zhao et al., 2016). This suggests that targeting improvement of mitochondrial dysfunction may be a therapeutic strategy for WC syndrome.

Among the marker genes for WC syndrome, the low expression level of IGHE may be related to the inhibition of B cell activation by viral infection (Tan et al., 2020). MT-ND5, MT-ND6, and MT-TE are important mitochondrial genes that show reduced expression on day 1, possibly stemming from mitochondrial dysfunction cleared by autophagy (Xu et al., 2020). As we know, fever is triggered by various pro-inflammatory cytokines that signal the hypothalamus to raise the body’s temperature set point, resulting in increased heat production by mitochondria (Eccles, 2005). Both pro-inflammatory cytokine pathways and thermogenesis pathways are activated in WC syndrome and WH syndrome. However, a pronounced sensation of chilliness specifically manifests in WC syndrome. This may be due to a passive elevation of the fever set point and a lack of mitochondrial productivity.

Sore throat is a prominent symptom of WH syndrome, associated with the release of inflammatory factors from the pharynx, which leads to vasodilatation, increased vascular permeability and sensitisation of sensory nerve endings (Kuchar et al., 2015). Immune infiltration analyses showed that monocytes, which are the main source of inflammatory factors such as TNF-α, IL-1 and IL-6, are more prevalent in WH syndrome. Among the marker genes for WH syndrome, G0S2 and FOSB are important pro-inflammatory genes (Wang et al., 2021; Binvignat et al., 2024). Both gene expression changes and symptomatic manifestations suggest that the inflammatory response is more intense in the wind-heat syndrome. Key inflammatory pathways such as TNFα/NFκB were significantly upregulated in both syndromes. However, the reason for the more intense inflammatory response in WH syndrome remains an intriguing question.

Downregulation of the TGF β signaling pathway and the Wnt/β-Catenin signaling pathway are important features of WH syndrome. The TGF-β signaling pathway exerts anti-inflammatory effects by inhibiting the production of inflammatory factors and promoting the secretion of anti-inflammatory factors (Massague and Sheppard, 2023). A study found that H5N1 influenza virus infection resulted in increased morbidity and mortality by inhibiting TGF-β (Carlson et al., 2010). The Wnt/β-catenin pathway inhibits inflammation by suppressing NF-κB activity (Liu et al., 2022). Curcumin, an extract of a traditional Chinese medicine, attenuates lung inflammation in model mice by activating the Wnt/β-catenin pathway (Yang et al., 2017). Downregulation of both pathways suggests that an imbalance in the regulation of the inflammatory response may be associated with a more intense inflammatory response in WH syndrome.

On day 1, immune infiltration analysis revealed that monocytes, as early initiators of inflammation (Guilliams et al., 2018), had a higher proportion in the WH syndrome, indicating a more intense inflammatory response. However, the proportion of M2 macrophage cells, which play a crucial role in anti-inflammatory response (Yunna et al., 2020), is lower in WH syndrome compared to WC syndrome. Although the difference may not be significant due to a small sample size, it still suggests that the anti-inflammatory response is suppressed in WH syndrome. A recent study found that Lianhua Qingwen capsules, which are suitable for the WH syndrome, can prevent LPS-induced acute lung injury by promoting M2 macrophage infiltration (Yan et al., 2022). Based on these findings, promoting anti-inflammatory responses may be a potential avenue for treating WH syndrome.

Cold syndrome and heat syndrome can also occur in other diseases. In the cold syndrome of chronic gastritis, transcriptome analysis revealed low levels of energy metabolism as a feature (Li et al., 2013), which is also consistent with the features of the wind-cold syndrome in this study. In the heat syndrome of rheumatoid arthritis, an active inflammatory response characterises the gene expression (Jiang et al., 2011). The downregulation of the inhibitory inflammatory response pathway found in this study may provide support for this active inflammatory response. Based on these findings, it can be inferred that the same pathological mechanism may exist in the same syndrome of different diseases.

Currently, the diagnosis of WC and WH syndromes relies on a series of different symptoms exhibited by patients. This requires doctors to conduct detailed inquiries and possess a certain level of diagnostic capability. This approach introduces a high degree of subjectivity, leading to potential biases and variability in diagnosis, which can affect accuracy and consistency. In contrast, our random forest diagnostic model, with its high accuracy and AUC, suggests that the identified marker genes can serve as reliable biomarkers for distinguishing WC and WH syndromes. This model offers a promising step toward objective and precise TCM syndrome diagnosis, reducing the subjectivity inherent in traditional methods and paving the way for more standardized clinical application.

This study also had some limitations. First, the sample size for each syndrome was limited and samples were only collected on days 1 and 6 after onset of illness. Thus, we employed GSEA for differential gene enrichment, which relies on the ranked abundance of gene expression changes rather than strictly defined differently expressed genes. This approach helps minimize potential biases on differential expression analysis that arise from a small sample size, providing a more robust and comprehensive overview of pathway involvement. Meanwhile, the short timeframe of sampling may not capture the full progression of URTIs, so including a longer follow-up period could provide more comprehensive insights into the dynamic changes in gene expression. Second, this study only described the different features of WC syndrome and WH syndrome at the transcriptome level, but did not explore the mechanism of the features. Further, the construction of the diagnostic model is of significant value, but due to the limited sample size, there is still a need to expand the sample size to guarantee the robustness of the diagnosis in future studies.

## Conclusion

This study revealed significant transcriptome-level differences between WC syndrome and WH syndrome. WC syndrome is characterized by mitochondrial dysfunction, whereas WH syndrome is marked by downregulation of the TGF-β signaling pathway and the Wnt/β-catenin pathway. Additionally, a diagnostic model with good performance was constructed using marker genes identified in WC and WH syndromes, contributing to the objectification and precision of traditional Chinese medicine diagnosis.

## Data Availability

The datasets presented in this study can be found in online repositories. The names of the repository/repositories and accession number(s) can be found below: https://github.com/hongyi27/Wind_Cold_VS_Wind_Heat, https://github.com/hongyi27/Wind_Cold_VS_Wind_Heat.
