# Supplementary material for: Longitudinal transcriptome analysis reveals distinct gene expression patterns in traditional Chinese medicine syndromes of upper respiratory tract infections
Source: Front Genet. 2024 Nov 26;15:1483098. doi: 10.3389/fgene.2024.1483098 (PMC11628533; doi:10.3389/fgene.2024.1483098)
Supplement: Supplementary file 2 [file Table1.docx]

Supplementary Material

# Supplementary Figures and Tables

## Supplementary Figures


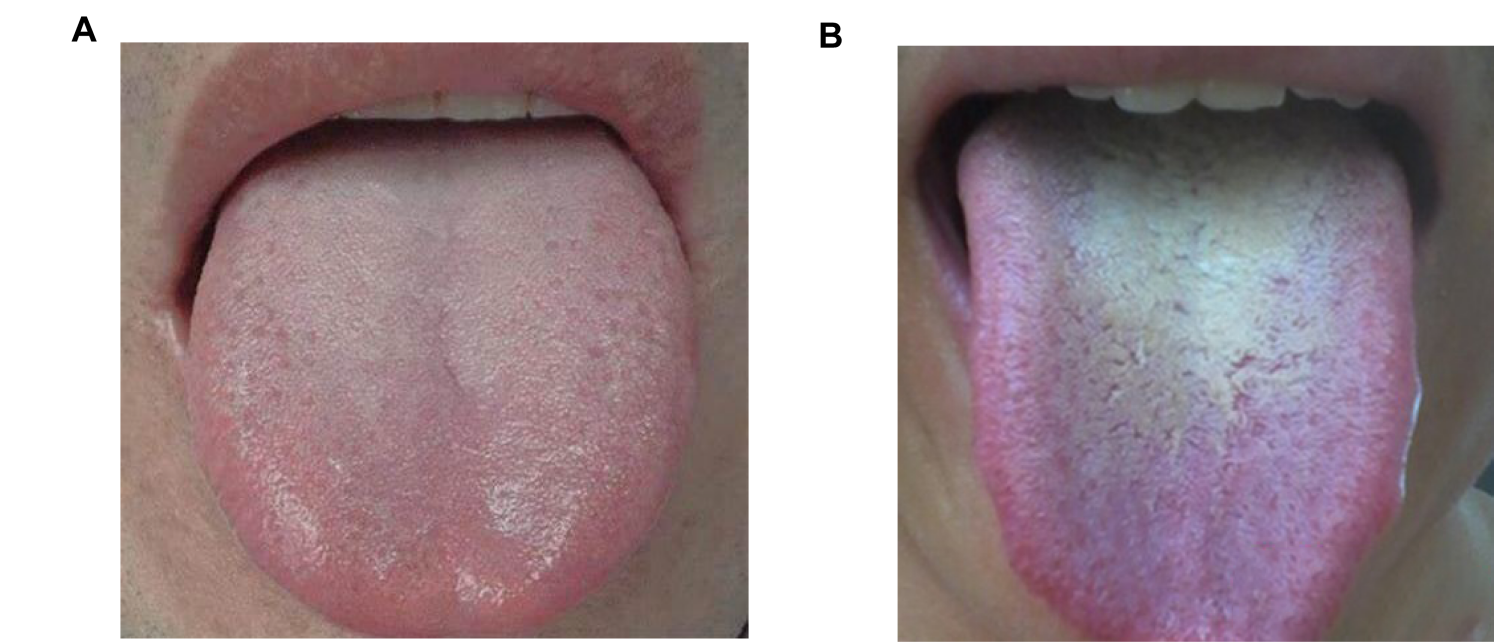


**Supplementary Figure 1.** Reference photos of tongue manifestations. (A) a thin white coating on the tongue. (B) a red tongue with thin yellow coating.


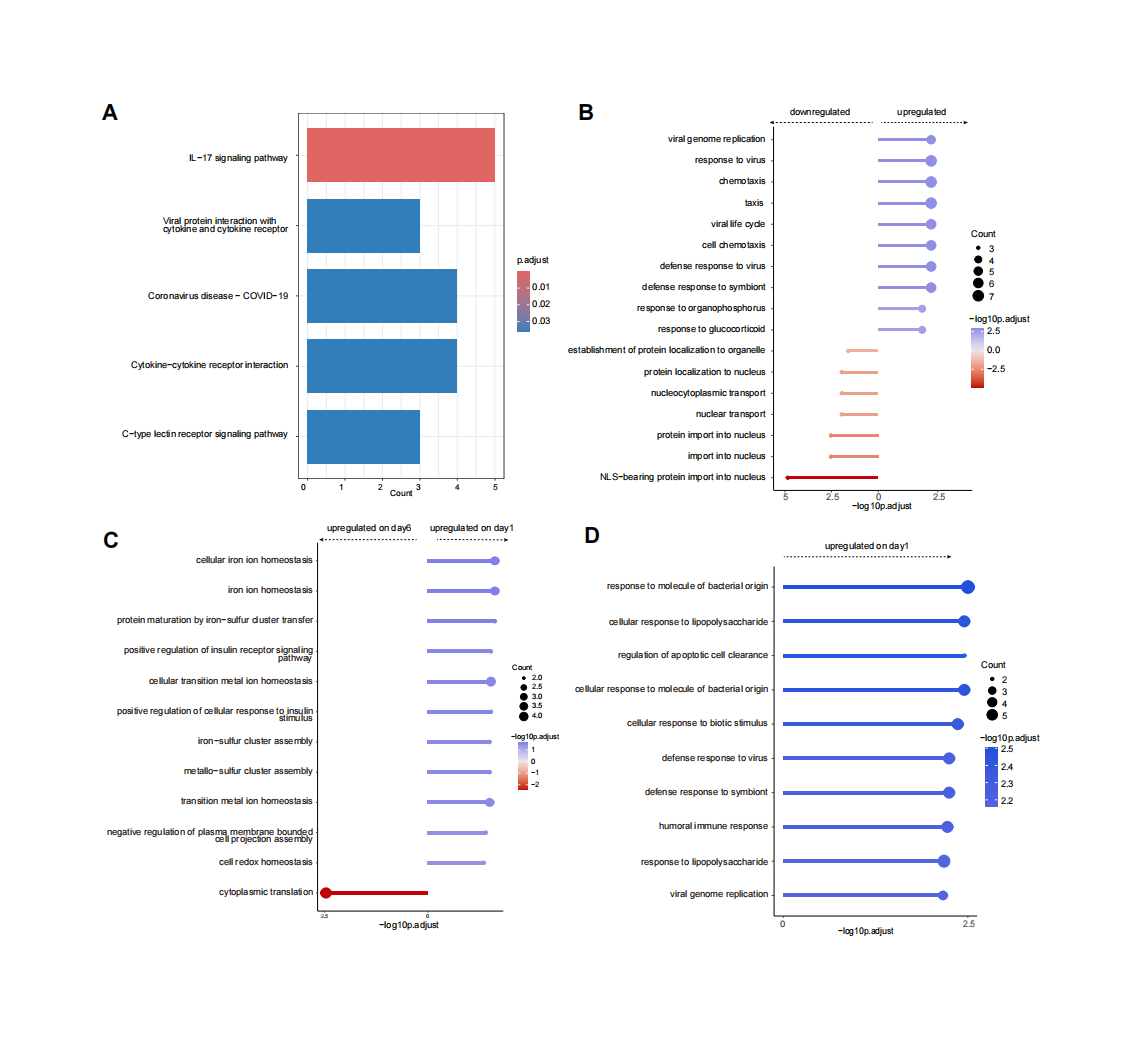


**Supplementary Figure 2.** Enrichment analysis of differentially expressed genes. (A)KEGG enrichment analysis of genes regulated in both WC and WH syndromes on day 1 compared to the healthy group. (B) GO enrichment analysis of genes regulated in both WC and WH syndromes on day 1 compared to the healthy group. (C) GO enrichment analysis of differentially expressed genes between day 1 and day 6 in WC syndrome. (D) GO enrichment analysis of differentially expressed genes between day 1 and day 6 in WH syndrome.

**
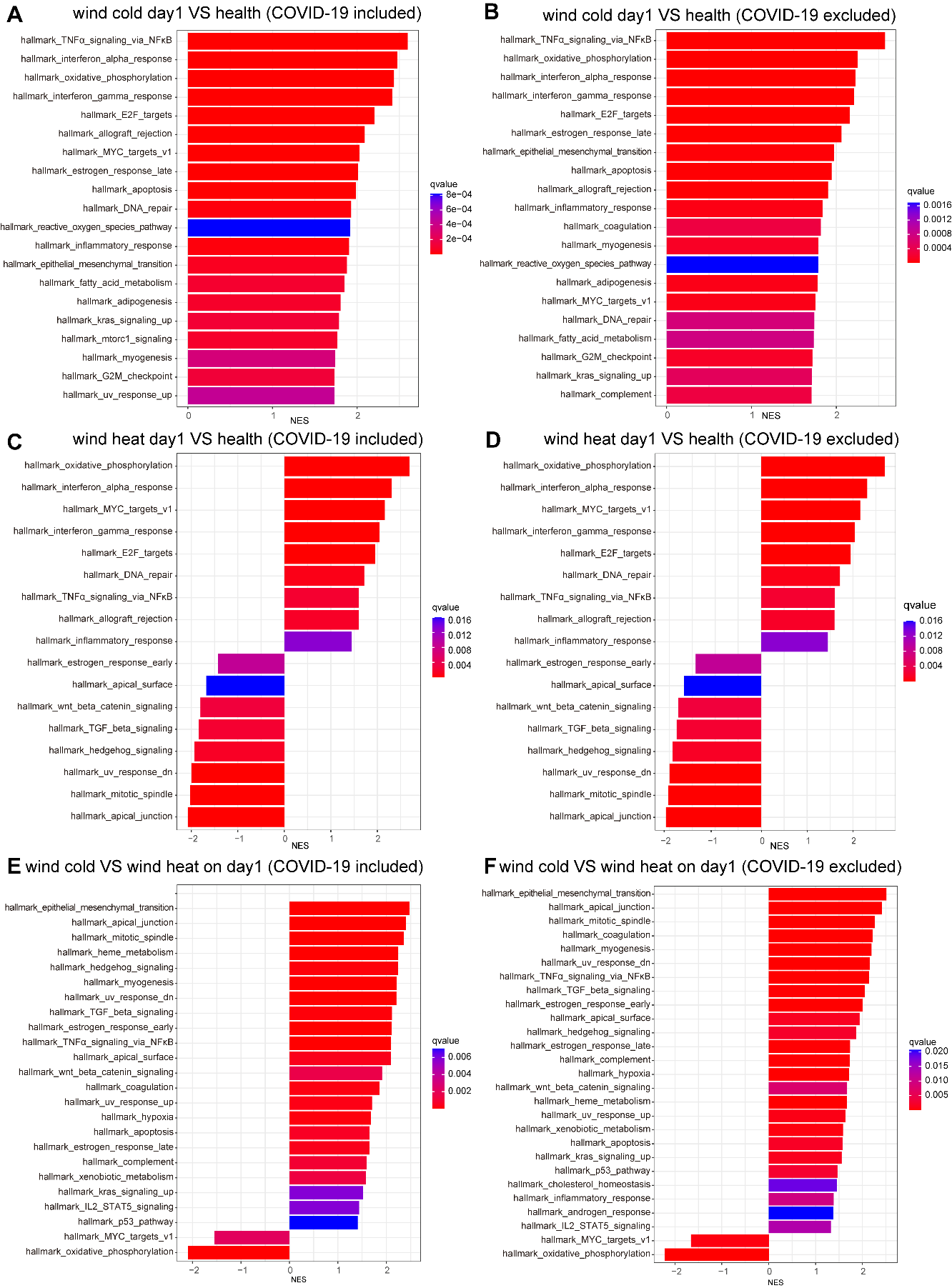
**

**Supplementary Figure 3.** Sensitivity analyses for COVID-19 samples. (A) top 20 pathway of GSEA of differentially expressed genes for WC vs healthy on day1, COVID-19 samples were included. (B) top 20 pathway of GSEA of differentially expressed genes for WC vs healthy on day1, COVID-19 samples were excluded. (C) GSEA of differentially expressed genes for WH vs healthy on day1, COVID-19 samples were included. (D) GSEA of differentially expressed genes for WH vs healthy on day1, COVID-19 samples were excluded. (E) GSEA of differentially expressed genes for WH vs WC on day1, COVID-19 samples were included. (F) GSEA of differentially expressed genes for WH vs WC on day1, COVID-19 samples were excluded.

**
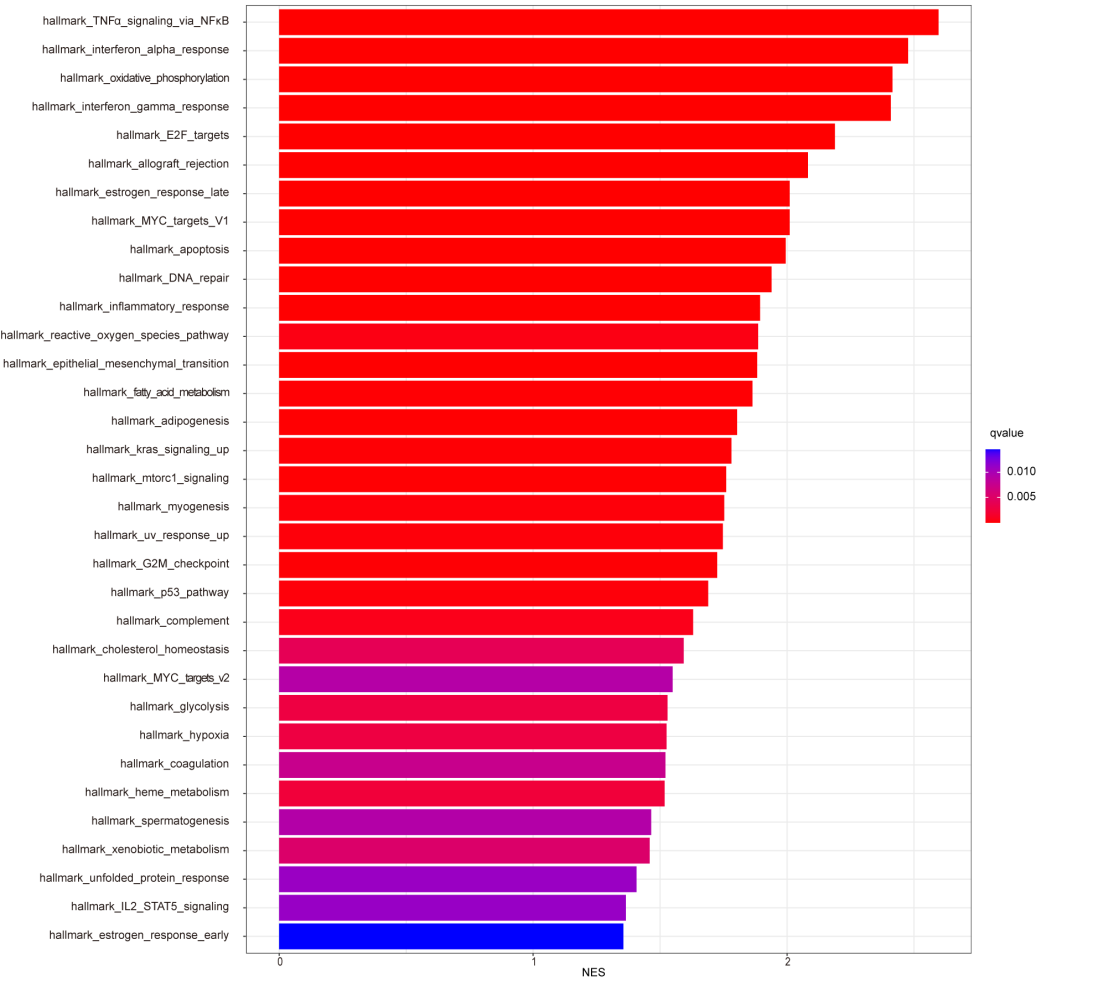
**

**Supplementary Figure 4.** All pathways analysed by GSEA of differentially expressed genes for WC vs healthy on day1.


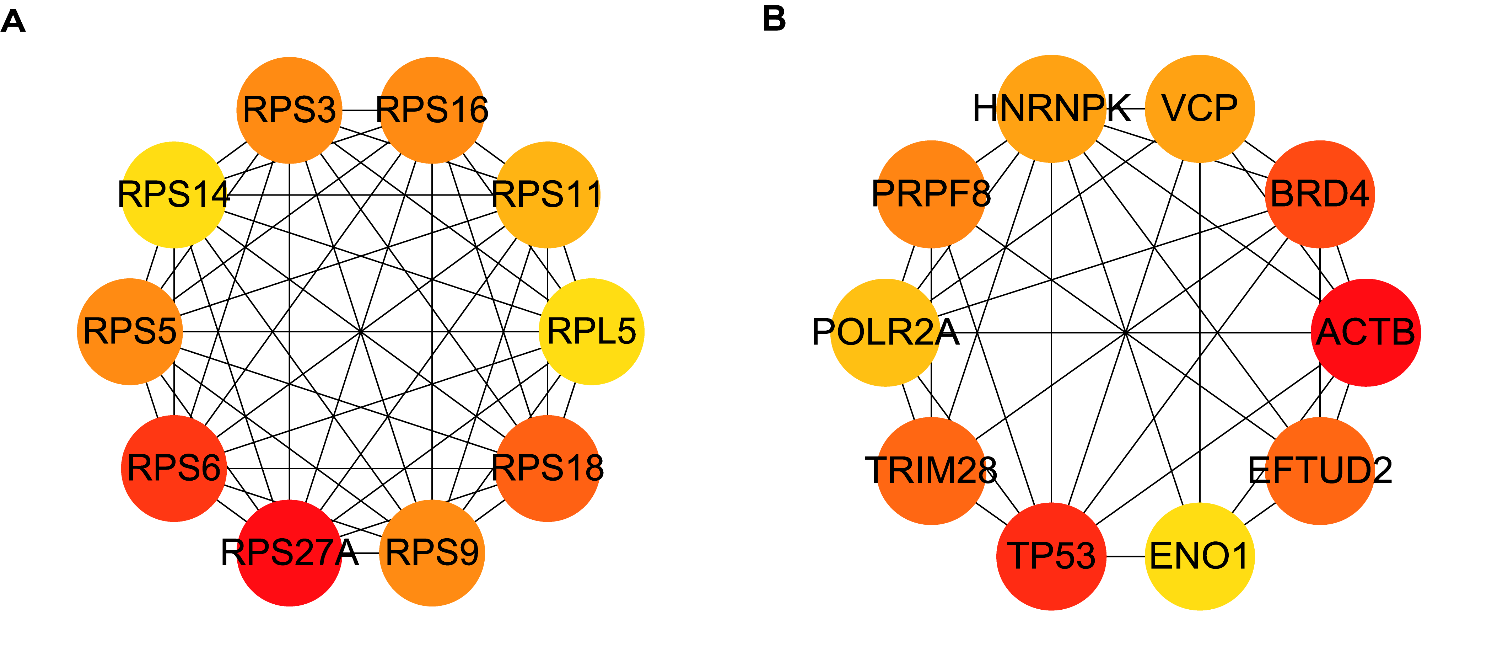
**Supplementary Figure 5.** Top ten hub genes in modules of interest. (A) Top ten hub genes within the brown, black, turquoise and red gene modules. (B) Top ten hub genes within the pink gene module.


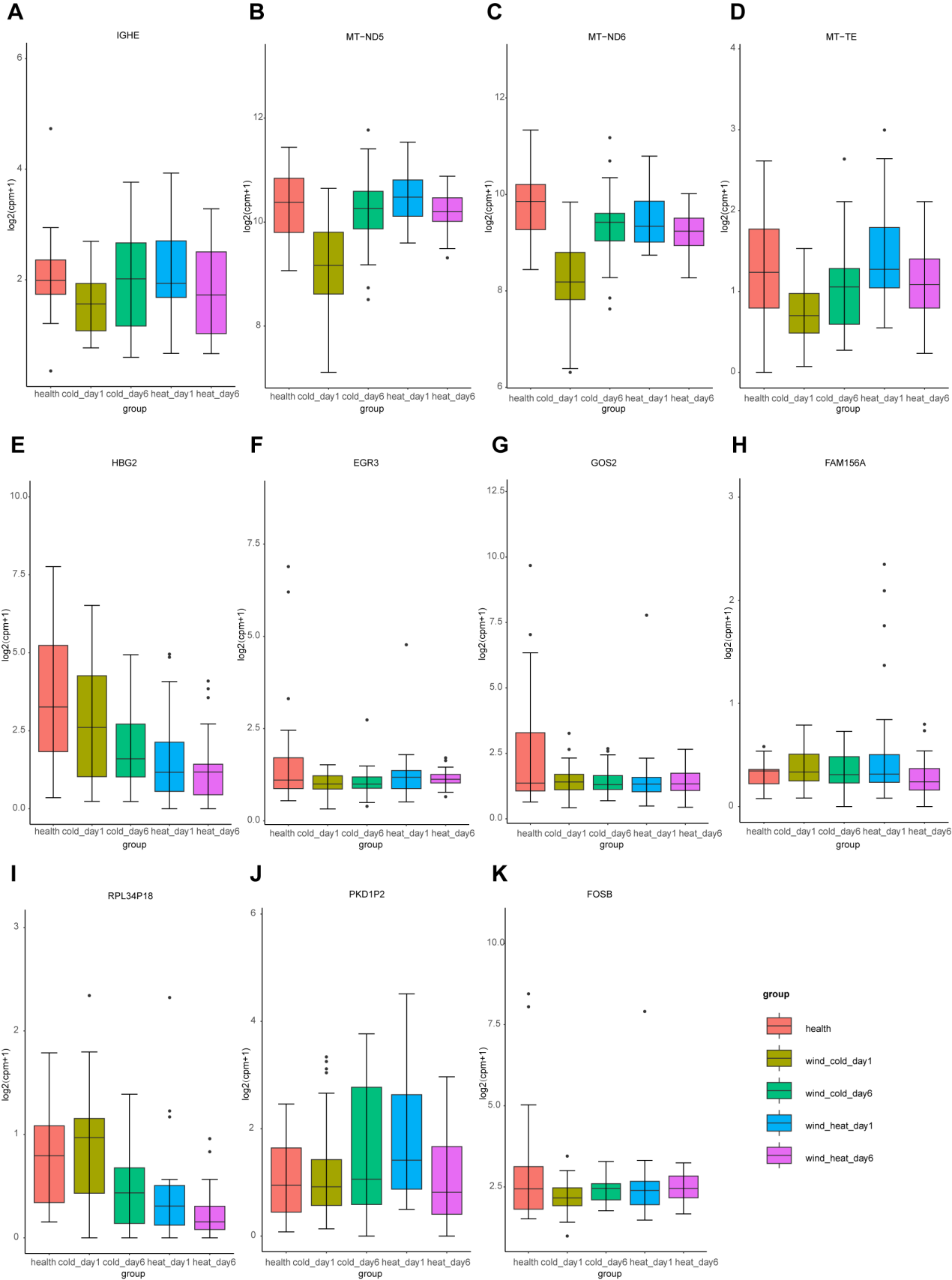


**Supplementary Figure 6.** Comparison of expression levels of marker genes among different groups. (A) Comparison of expression levels of IGHE among different groups. (B) Comparison of expression levels of HBG2 among different groups.(C) Comparison of expression levels of MT-ND5 among different groups. (D) Comparison of expression levels of MT-ND6 among different groups. (E) Comparison of expression levels of MT-TE among different groups. (F) Comparison of expression levels of EGR3 among different groups. (G) Comparison of expression levels of GOS2 among different groups. (H) Comparison of expression levels of FAM156A among different groups. (I) Comparison of expression levels of RPL34P18 among different groups. (J) Comparison of expression levels of PKD1P2 among different groups. (K) Comparison of expression levels of FOSB among different groups.

## Supplementary Tables

**Table S1** Upper Respiratory Tract Infection Symptom Scoring Sheet

| **Symptom** | **0 points** | **1 point** | **2 points** | **3 points** | **Scoring** |
| --- | --- | --- | --- | --- | --- |
| Fever | Temperature (Axillary) ≤37.2℃ | Temperature (Axillary) 37.3℃～38.5℃ | Temperature (Axillary) 38.6℃～39.5℃ | Temperature (Axillary) >39.5℃ |  |
| Headache or muscle aches | None | Mild pain, intermittent | Sustained pain, but tolerable | Severe pain, intolerable |  |
| Sore throat | None | Sore throat, but does not affect swallowing | Dryness and mild burning sensation in the throat, mild pain when swallowing | Severe pain when swallowing, noticeable burning sensation |  |
| Fatigue | None | Slightly fatigued, but does not affect daily life and work | Fatigued, affecting daily life and work | Extremely fatigued, no desire to engage in any activity |  |
| Runny nose | None | Slight runny nose, easily tolerable | Noticeable runny nose, annoying but bearable | Unbearable runny nose, affecting daily life or sleep |  |
| Nasal obstruction | None | Slight nasal congestion, speaking with a nasal tone | Noticeable nasal congestion, sometimes relieved | Persistent and unrelieved nasal congestion |  |
| Sensation of chilliness | None | A slight chill sensation and sensitive to wind | A conspicuous sensation of chilliness, adding more clothes does not relieve it | Shivering |  |

**Table S2** Comparison with results of transcriptome analyses of other upper respiratory tract infections.

| References | Object | Pathways of differential gene enrichment |
| --- | --- | --- |
| (Rosa et al., 2021) | SARS-CoV-2 positive  VS negative | Neutrophil degranulation pathway upregulated  **IFN signaling pathway upregulated**  Innate immune system pathway upregulated  Collagen degradation pathway downregulated  **TGFβ signaling pathway downregulated** |
| (Herberg et al., 2013) | H1N1/09 influenza VS health control | **Inflammatory response pathways upregulated**  **Interferon signaling pathways upregulated**  Protein synthesis-related pathways downregulated |
| (Besteman et al., 2020) | cases of respiratory syncytial virus infection VS cases without lung infection | **NF-kB signaling upregulated**  **IL-6 pathways upregulated**  **interferon pathways upregulated**  Neutrophil activation gene expression upregulated |
| (Tang et al., 2022) | SARS-CoV-2 infection VS health control | Immunoglobulin complex pathway upregulated  **Sensing response to bacteria pathway upregulated**  B-cell receptive signaling pathway upregulated  Glycolysis/gluconeogenesis pathway upregulated  Purine metabolism pathway upregulated  **Electron transport chain pathway upregulated** |
| (Zhou et al., 2020) | COVID-19 VS healthy controls | **Interferon pathway upregulated**  **Chemokine signaling pathway upregulated**  **IL-17 signaling pathway upregulated**  **TNF signaling pathway upregulated**  **NF-κB signaling pathway upregulated** |
| (Guarnieri et al., 2023) | SARS-CoV-2 positive VS negative | **Oxidative phosphorylation pathway downregulated**  Glycolytic pathway upregulated |
| (Nicoletti et al., 2022) | severe/critical patients with COVID-19 VS mild patients with COVID-19 | **Wnt/β-catenin signaling pathway downregulated**  STAT3 signaling pathway downregulated |

Pathways bolded in the table showed consistent changes in this study, pathways not bolded were not found in this study.
