# Supplementary figures and images for: Longitudinal transcriptome analysis reveals distinct gene expression patterns in traditional Chinese medicine syndromes of upper respiratory tract infections
Source: Front Genet. 2024 Nov 26;15:1483098. doi: 10.3389/fgene.2024.1483098 (PMC11628533; doi:10.3389/fgene.2024.1483098)

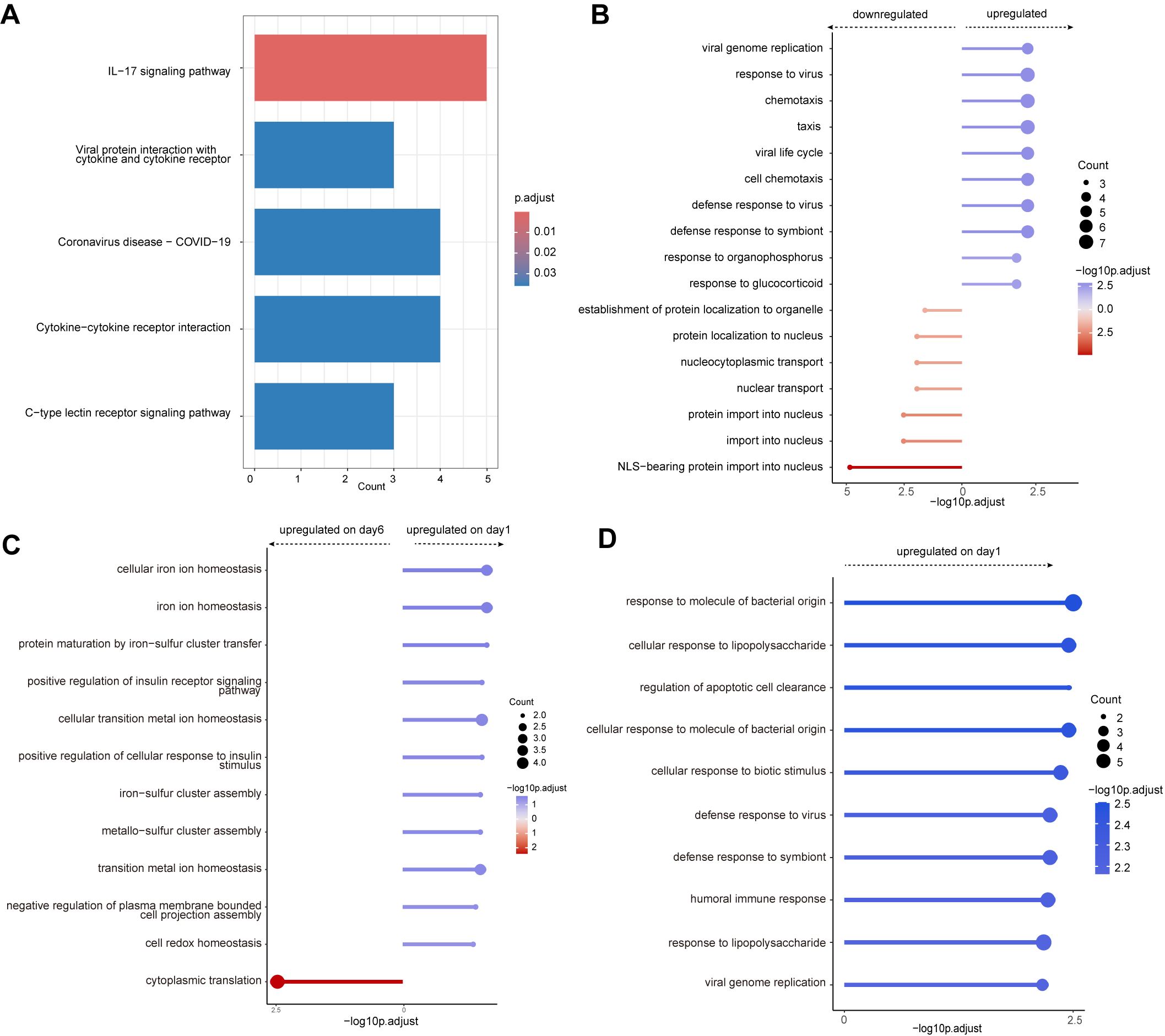

Supplement: Supplementary file 3 [file Image1.tif]
